# Intragraft regulatory T cells in the modern era: what can high-dimensional methods tell us about pathways to allograft acceptance?

**DOI:** 10.3389/fimmu.2023.1291649

**Published:** 2023-11-23

**Authors:** Ke Fan Bei, Sajad Moshkelgosha, Bo Jie Liu, Stephen Juvet

**Affiliations:** ^1^ Latner Thoracic Research Laboratories, Toronto General Hospital Research Institute, University Health Network, Toronto, ON, Canada; ^2^ Department of Immunology, University of Toronto, Toronto, ON, Canada; ^3^ Toronto Lung Transplant Program, Ajmera Transplant Centre, University Health Network, Toronto, ON, Canada

**Keywords:** regulatory T cells (Treg), organ transplantation, intragraft, regulatory T cell migration, single cell RNA sequencing, (allograft) function/dysfunction, high-dimensional methods

## Abstract

Replacement of diseased organs with transplanted healthy donor ones remains the best and often only treatment option for end-stage organ disease. Immunosuppressants have decreased the incidence of acute rejection, but long-term survival remains limited. The broad action of current immunosuppressive drugs results in global immune impairment, increasing the risk of cancer and infections. Hence, achievement of allograft tolerance, in which graft function is maintained in the absence of global immunosuppression, has long been the aim of transplant clinicians and scientists. Regulatory T cells (Treg) are a specialized subset of immune cells that control a diverse array of immune responses, can prevent allograft rejection in animals, and have recently been explored in early phase clinical trials as an adoptive cellular therapy in transplant recipients. It has been established that allograft residency by Tregs can promote graft acceptance, but whether intragraft Treg functional diversification and spatial organization contribute to this process is largely unknown. In this review, we will explore what is known regarding the properties of intragraft Tregs during allograft acceptance and rejection. We will summarize recent advances in understanding Treg tissue residency through spatial, transcriptomic and high-dimensional cytometric methods in both animal and human studies. Our discussion will explore properties of intragraft Tregs in mediating operational tolerance to commonly transplanted solid organs. Finally, given recent developments in Treg cellular therapy, we will review emerging knowledge of whether and how these adoptively transferred cells enter allografts in humans. An understanding of the properties of intragraft Tregs will help lay the foundation for future therapies that will promote immune tolerance.

## Introduction

Solid organ transplantation can improve survival and quality of life for those with end-stage organ disease. Originally a risky procedure with short-term success due to acute rejection, over the decades this has changed dramatically with advances in medicine and an improved understanding of immunology. Early post-transplant mortality has now decreased due to the development of immunosuppressants. Several immunosuppressive drugs are commonly used in tandem to prevent the rejection of the graft(1). However, transplant is still not a cure due to the eventual failure of the transplanted organ from chronic rejection and fibrosis as a result of the alloimmune response. Furthermore, broad suppression of the immune system over a long period of time leads to many serious side effects including nephrotoxicity, as well as increased risks for diabetes, cancer and infections, limiting long-term survival (1). Hence, achievement of tolerance, a state of non-responsiveness to donor antigen with preserved protective immunity, has been a major goal of transplant clinicians and scientists. Regulatory T cells (Tregs), which have the ability to maintain immune homeostasis and prevent autoimmunity, have been the focus of many attempts to manipulate the immune system toward operational tolerance, defined as maintenance of graft without the need for immunosuppression for over 12 months with otherwise normal immunity (2).

## Definition and properties of Tregs

The existence of T cells with suppressive properties was suspected for many years, but definitive identification was not possible until CD4^+^CD25^+^ T cells were shown to mediate allograft tolerance (3, 4). Later the now well-known transcription factor, Forkhead Box P3 (FOXP3) was discovered to be essential to the differentiation and function of this regulatory T cell population (5, 6). In humans, the recognition of immune dysregulation, polyendocrinopathy, enteropathy, X-linked syndrome (IPEX) as an autoimmune disorder arising due to FOXP3 mutations, solidified FOXP3 as the key transcription factor in Tregs (7, 8). Canonically CD4^+^CD25^+^FoxP3^+^ Tregs account for approximately 5-10% of CD4^+^ cells (9), and are now recognized to be a heterogeneous population with transcriptionally distinct subsets (10, 11)). Tregs have multiple methods of regulating immune responses. First, they produce the inhibitory cytokines interleukin 10 (IL-10), interleukin 35 (IL-35) and transforming growth factor β (TGF-β), which suppress nearby effector T cells (12–15). Additional inhibitory small molecules produced by Tregs include adenosine (16–18) and Cyclic adenosine monophosphate (cAMP) (19). Cytolytic Tregs kill effector T and B cells through the release of granzymes and activation of apoptosis through TRAIL (20, 21). In addition, as Tregs are unable to produce their own IL-2 and require IL-2 to survive; they compete for it with other effector T cells, thus depriving the latter of this essential cytokine (14, 15). Finally, Tregs can modulate dendritic cell function via cytotoxic T lymphocyte antigen 4 (CTLA4)-B7 interaction (22) which mediates inhibitory signaling in dendritic cells (DCs) preventing their maturation (23, 24). Tregs have been shown to limit the interaction between DCs and naïve CD4 T cells *in vivo* via two-photon microscopy (25). Tregs can also limit priming of T cells by DCs through the CTLA4-B7 interaction (26). Further, Tregs can use CTLA4 to remove B7 molecules from the DCs surface, reducing their costimulatory function (27). The multiple modes of suppression and inhibition at their disposal render Tregs an interesting therapeutic candidate to prevent acute and chronic allograft rejection.

More recently, in addition to CD25 and FOXP3, low interleukin-7 receptor (CD127) expression has been used to further distinguish Tregs from other helper T cell populations (28–30). Other markers are also now being used to delineate Tregs from T cells, including co-stimulatory markers (ICOS, OX40, GITR, 4-1BB), co-inhibitory markers (CTLA-4, TIGIT, LAG3), and trafficking molecules (CXCR4, CXCR5, CCR4, CCR5, CCR7, CCR8 and CXCR7) (31), while expression of these markers is not limited to Tregs, their expression pattern and level is distinct from the majority of conventional T cells (31). In mouse, in addition to the common Treg markers, the surface marker neuropilin-1 (Nrp1/CD304) has been linked to Treg interaction with dendritic cells, although its expression on human Tregs has been uninformative (32). In the absence of inflammation, Nrp1 mediated prolonged Treg interactions with immature DCs leading to homeostatic suppression of immune response (33). In addition, Weiss et al., described Nrp1 as a marker of murine thymic Tregs (tTregs) under noninflammatory conditions but is also expressed on peripheral Tregs (pTregs) in highly inflamed environments (34). Glycoprotein A repetitions predominant (GARP), which is a receptor for latent TGF-β, is expressed on activated Tregs with immune suppressive potential (32, 35).

CD39 is an ectoenzyme involved in adenosine synthesis (18); its expression has been correlated with FOXP3 stability in human Tregs and protection from xenograft versus host disease in mice (36). The transcription factor Helios is correlated with human Treg stability but does not mediate stability; Treg function and stability were equivalent between Helios-deleted Tregs and unedited Tregs (37). These studies highlight the dynamics of Tregs and that to adequately distinguish Tregs from conventional T cells, a combination of surface markers and transcription factors needs to be considered.

The development of Tregs occurs via two distinct pathways. tTregs are generated in the thymus and later migrate to the periphery while pTregs arise directly in the periphery through differentiation from naïve FOXP3^-^CD4^+^ T cells (38). pTregs have been found in the gut and the placenta where they modulate interactions with the external environment – including the microbiota – and prevent aberrant antigen responses. Tregs can also be induced *in vitro* through induction of conventional T cells with TGF-β and interleukin 2 (IL-2) known as induced Tregs (iTregs) (38). The degree of demethylation of the Treg-specific demethylated region (TSDR), a non-coding element in the FOXP3 locus, differs between tTregs and iTregs. A relatively lower degree of TSDR demethylation in iTregs renders them unstable in FOXP3 expression and immunomodulatory function (8, 39). For pTregs, induction by TGF-β in the periphery and binding of the Foxp3 conserved noncoding sequence 1 (CNS1) by NFAT is required for differentiation. Furthermore, although FOXP3 is the main transcription factor driving Treg phenotype, the expression of FOXP3 in Tregs requires T cell receptor (TCR) signaling, where the recognition of self-antigens presented by major histocompatibility complex (MHC) by the TCR is an essential step in tTreg development (40). Described as a “goldilocks principle”, strong and transient TCR stimulation is needed for Treg cell lineage commitment and robust FOXP3 expression in CD4^+^ single positive thymocytes, whereas a weak signal leads to conventional T cell development and a persistent strong signal results in negative selection (41). This is also true for iTregs, where a strong TCR signal is needed for induction (41). Naïve T cells that encounter low amounts of high affinity agonist antigens resulted in FOXP3 expression *in vivo*, creating pTregs (42). Furthermore, activation of Treg suppressive function is TCR-dependent. Only after activation can TCR-non-specific suppression of bystander cells occur (40). The development of pTregs has been likened to that of iTregs, but it should be noted that pTregs are induced *in vivo* where other microenvironmental factors may be a driving factor (38). Whether such mechanisms are similarly in place in allografts, and the proportional involvement of tTregs and pTregs in allograft tolerance are currently unknown as markers that reliably distinguish the two populations in humans are unclear (43).

## Tregs must enter allografts to enable graft acceptance

In animal models, allograft tolerance has been shown to be dependent on recipient Treg function and survival. Animal studies in which Tregs were depleted through either thymectomy or a CD25 monoclonal antibody demonstrated abrogation of allograft acceptance, resulting in rejection (44, 45). Tolerance mediated by Tregs is donor specific, as second donor-specific allografts were accepted, but third-party grafts were rejected indicating that this was not a result of generalized immune defects or broad suppression of the immune system (44). In animal models, occupancy of the allograft by T cells with regulatory function has been shown to be required for allograft tolerance (46). In a mouse skin transplant model treated with non-depleting CD4 and CD8 monoclonal antibodies to induce tolerance, re-grafting of tolerated skin grafts into thymectomized and T-cell depleted host mice prevented rejection when fresh naïve splenocytes were infused (46). The re-transplanted allograft was found to contain T cells that exited the graft, expanded and colonized the new host to an extent that was detectable in the peripheral blood; further, they prevented the rejection of fresh donor-specific allograft, suggesting the presence of intragraft T cell populations including CD4^+^CD25^+^ Tregs that drive transferrable, dominant tolerance (46). A similar observation was made in a murine pancreatic islet study in which Tregs maintained stable graft function, and transfer of intragraft Tregs into the newly transplanted graft resulted in induction of tolerance (47). However, FOXP3 mRNA expression in allografts has also been associated with acute rejection; in heart transplant recipients, Tregs could be isolated from endomyocardial biopsies from patients with acute cellular rejection and expanded in culture (48). Furthermore, even though persistence of donor antigens is required for tolerance in some models, this may not be sufficient. In a murine model of skin transplant tolerized by IL-2-IL-2 antibody complex treatment, Tregs were not able to drive tolerance of a second allograft but instead led to rejection of the primary graft, breaking established tolerance (49). This observation suggested that in this model, intragraft Tregs prevented rejection but were not able to exit the graft and expand sufficiently to control the rejection of a second donor graft. The mode and timing of tolerance induction, strain differences, or alterations within intragraft Treg populations may be a factor in development of tolerance, as induction of peripheral Tregs from FOXP3 deficient T cells through TGF-β signaling promoted skin graft survival in mice treated with a non-depleting anti-CD4 antibody, and were able to accept a second fresh graft after over 100 days of the initial treated graft (50).

Accumulation of intra-graft FoxP3-expressing cells has been observed in biopsies of liver allografts in operationally tolerant patients. In a study of 69 adult liver transplant recipients where 14 proceeded to biomarker-guided immunosuppression withdrawal and 8 achieved operational tolerance, the ratio of intrahepatic FOXP3 to IFNG gene expression was elevated in tolerant patients prior to immunosuppression withdrawal, suggesting that intragraft Tregs mediated this state of tolerance (51). The gene for E-selectin, an adhesion molecule responsible for lymphocyte recruitment, was higher in liver biopsies of tolerant patients, which might reflect an enhanced ability of these grafts to retain Tregs (51). Furthermore, accumulation of CD4^+^FOXP3^+^ cells was found to occur after immunosuppression withdrawal followed by downregulation of proinflammatory genes in liver transplant patients without progressing to rejection (52). Similarly, subclinical rejection – but not acute rejection – of liver allografts was associated with higher ratio of CD4^+^FOXP3^+^ Tregs to CD8^+^ T cells (53). In addition, Tregs can be found in allografts many years after transplantation. For example, FOXP3 expression was identified in a human composite vascularized allograft up to six years post-transplant; TCR-Vβ repertoire analysis indicated that a select subset of presumably alloreactive Tregs were expanded (54). In contrast, some studies have associated Tregs and FOXP3 expression with adverse graft outcomes. In a kidney transplant study of 36 individuals with acute rejection, 18 with chronic allograft nephropathy and 29 with normal biopsies, urine mRNA levels of FOXP3 were predictive of graft failure within six months of an acute rejection episode (55). Therefore, whether the presence of FOXP3 is protective or detrimental to the survival of the allograft is unclear. One factor to consider is that FOXP3 expression on its own is not synonymous with a regulatory phenotype in CD4^+^ T cells. FOXP3 expression has been detected in CD4^+^CD25^-^ T cells in systemic lupus erythematosus patients (56). Other cells types such as activated CD4^+^CD25^-^T cells have also shown transient expression of FOXP3 without suppressive capabilities (57) as well FOXP3 has also been described in certain cancers (58, 59). Another possibility to consider is that altered patterns of Treg activation and migration into grafts may tip the balance toward either protection or injury.

**Figure 1:**
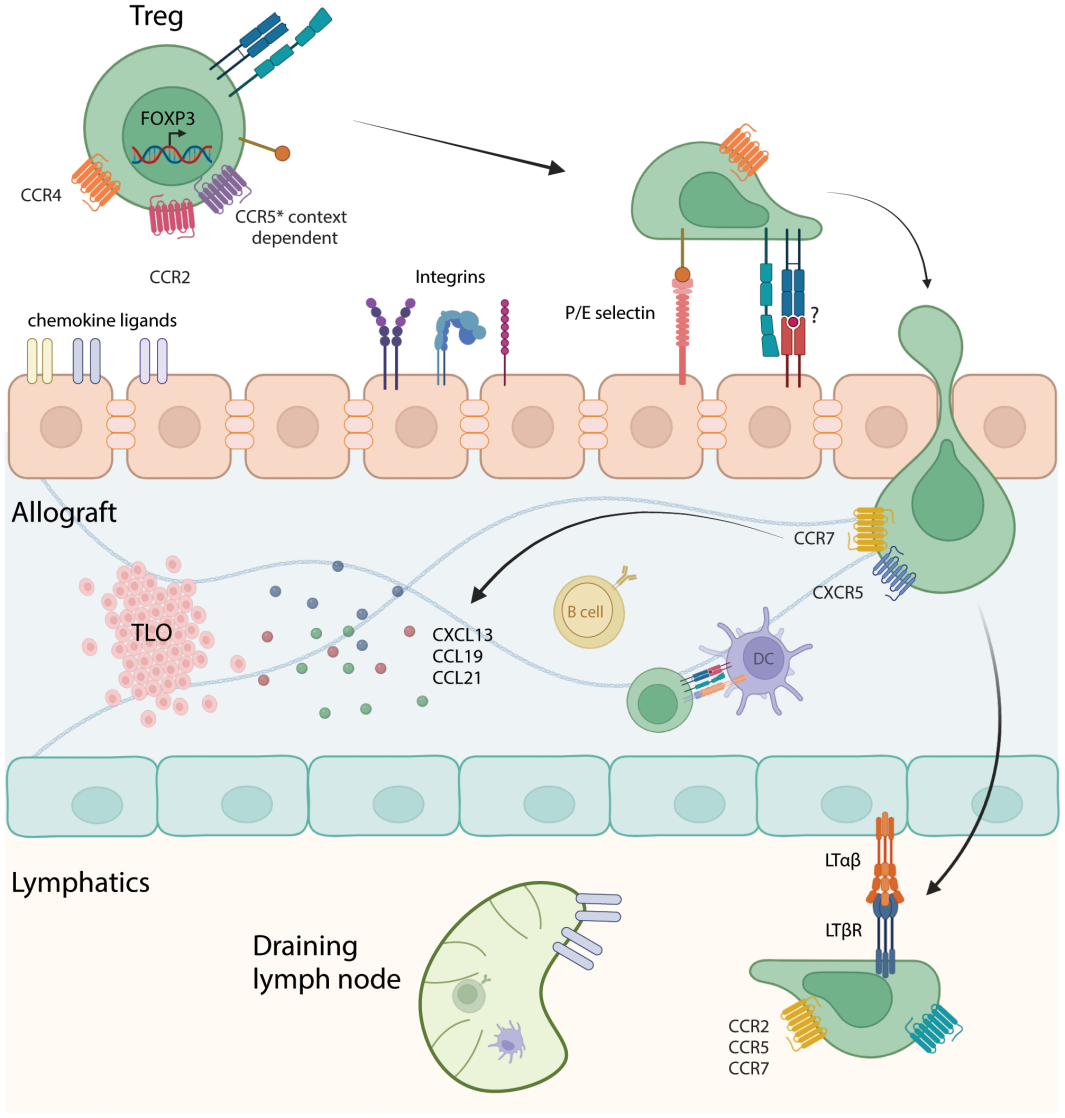
Interaction of Tregs with the vascular endothelium, intragraft cells, and lymphatics. Depiction of Tregs entering the allograft through interactions with chemokine ligands, integrins and potentially through TCR-MHC binding. Diapedesis of Tregs into the allograft, encountering antigen-presenting cells such as B cells and dendritic cells, which can prime and sustain anti-donor T cell responses. Migration to tertiary lymphoid organs (TLOs) can occur through interactions with chemokines such as CXCL13, CCL19 and CCL21. Subsequently, Tregs enters the lymphatics and migrate to the draining lymph node through LTαβ/LTβR interaction and based on chemokine receptors CCR2, CCR5 and CCR7. Made using Biorender.com.

Investigators have sought to identify molecules involved in Treg homing to allografts (**Figure 1**). In a murine cardiac transplant study, recruitment of Tregs into the allograft was dependent on chemokine receptor CCR4 and macrophage-derived chemokine (CCL22) whereas in the absence of Treg recruitment into the graft, tolerizing therapies were ineffectual (44). CCL5 is chemokine with three receptors: CCR1, CCR3 and CCR5 (60). These receptors mediate recruitment leukocytes to sites of inflammation. CCL5 mediated Treg trafficking into rat kidney allografts (61). The homing of Tregs sequentially to the allograft first via P/E-selectin, CCR2, CCR4 and CCR5 and then to the draining lymph node using CCR2, CCR5 and CCR7 is required for activation and immune modulation (62). Further work showed that the migration of Tregs through graft lymphatics is required to regulate allogeneic T cells in the draining lymph nodes, a process that involves the interactions between lymphotoxin β receptor(LTβR) presented by lymphatic endothelial cells to LTαβ expressed on Tregs (63). In contrast, chemokines may have negative effects on Treg recruitment to the allograft, as tolerant grafts had high CCR4 expression on Tregs while rejected grafts had high CCR5 and CXCR3 expression on Tregs (44).

Activated Tregs target effector T cells, preventing their proliferation and migration to the graft, as well as inhibiting donor-derived dendritic cell migration (62). Trafficking of Tregs into allogeneic tissues may also be dependent on major histocompatibility complex molecules or in the case of humans, human leukocyte antigens (HLA). Development of donor-specific human chimeric antigen receptor Tregs (CAR-Treg) against a class I HLA molecule, HLA-A2, was found to direct trafficking of CAR-Tregs into the A2 expressing allograft over nonspecific control in a mouse skin transplant model (64). Transfer of these A2-CAR Tregs suppressed T cell activation and subsequently rejection of a skin allograft (64). These A2-transduced CAR Tregs exhibited better control of allospecific immune responses and completely prevented rejection *in vitro* and *in vivo* in a humanized skin transplant model, without affecting the basic stability and function of the Tregs as demonstrated by Noyan et al. (65). Therefore, Treg and HLA specificity is likely to be needed for Treg homing and function, although how HLA class II molecules interact with Treg trafficking is not fully understood. These data suggest that directed migration of Tregs to the graft is critical to their effectiveness of immune modulation and at controlling of anti-donor responses.

Interestingly, the programmed death 1 (PD-1)/programmed death ligand 1 (PD-L1) pathway, which has various roles in T cell function, has also been investigated in Treg function. Tregs can use PD-L1 to suppress autoreactive B cells in a mice expressing model antigens in the kidney (66). In the transplant context, a rat model of kidney transplant using membrane-anchored-protein PD-L1 on glomerular endothelial resulted in higher FOXP3 expression in treated grafts (67). In a scRNAseq study of mouse pancreatic islet transplantation, expression of PD-1 on Tregs was associated with tolerance (68). How this mechanism operates to promote tolerance in allografts has not been well-defined and is an area for further research.

Various methods have been used to generate Treg cell therapy products for solid organ transplantation. In a 2016 pilot study in living donor liver transplantation, Tregs were generated in a 2-week culture with recipient and irradiated donor lymphocytes obtained from leukapheresis with CD80 and CD86 monoclonal antibodies to block costimulation (69); the resulting cells were administered, resulting in 7 of 10 recipients becoming operationally tolerant. Other studies differed in their methods of Treg manufacturing; approaches have included fluorescence activated sorting and *ex vivo* polyclonal expansion using stimulation beads (70) or depletion and enrichment using a CliniMACS-based system (71–73), which magnetically isolates CD25^+^ cells from CD8 and CD19-depleted peripheral blood mononuclear cells (73, 74). One of the most pivotal Treg therapy cell trials to date, the ONE study, was performed in living donor kidney transplantation. It was an international study involving France, Germany, Italy, the UK, and the USA (75). Six different cell therapy products were generated, four of which were Treg based (75) and included polyclonal Tregs (2 centers), Tregs generated via costimulatory blockade in the presence of donor peripheral blood mononuclear cells (one center), and Tregs stimulated with donor B cells activated by K562 expressing human CD40L (one center) (75). The study was underpowered to show clear differences in Treg products, but it preclinical studies show that polyclonal Tregs have lower potency than donor alloantigen-reactive Tregs (reviewed in (76)). It is also likely that genetic differences between individuals, pre-existing disease and other factors impact the number and quality of Tregs generated. For example, in the ARTEMIS study in liver transplantation, donor-antigen reactive Tregs (darTregs) were generated for cell therapy but many participants’ Tregs failed to expand sufficiently (77). Further work is needed to determine the factors that impact successful expansion of Tregs for use in clinical trials and their ability to control anti-donor immune responses within the graft. Knowledge of the properties of intragraft Tregs from preclinical studies will help to inform this effort.

## Tertiary lymphoid organs as sites of Treg-mediated immune regulation

In transplantation, intragraft lymphocytes have been observed to cluster and organize into ectopic lymphoid structures, to differentiate them from conventional primary and secondary lymphoid organs which are generally formed during embryogenesis. These ectopic structures arise in response to chronic inflammation, a common occurrence in transplant, in a process known as lymphoid neogenesis enabling localized immune response (78). They are therefore a potentially important microenvironment for graft infiltrating Tregs, and were first described in cardiac allograft biopsies in 1985 (79). The composition of these structures varies, but they generally have defined borders and are composed of lymphocytes with or without high endothelial venules (HEVs) – lined by specialized peripheral node addressin (PNAd)-expressing endothelial cells (80)– that enable lymphocytes to enter non-lymphoid tissue (78). Those with HEVs are often referred to as tertiary lymphoid organs (TLOs). It remains controversial whether these lymphoid structures promote or inhibit allograft survival (78, 81, 82). Due to inflammation-driven expression of stromal chemokines CXCL13, CCL19 and CCL21, T and B cells are recruited into allografts (78). Coupled with expression of tumor necrosis factor α (TNFα) and lymphotoxin-mediated activation of lymphotoxin β receptors on stromal cells, HEVs form and T and B cells segregate into distinct compartments (78).

In mouse cardiac allografts, a higher number of ‘Quilty lesions’ or TLOs were found in allografts that were chronically rejected compared to those with acute rejection suggesting that TLOs drive consistent immune modulation (83). Expression of FOXP3 has been described in these structures but its relation to allograft tolerance has not been established, likely due to the varying cellular composition of TLOs in different contexts. The presence of Tregs in TLOs was associated with higher cardiac allograft acceptance while in another mouse study, they were instead associated with acute and chronic rejection (78). In lung transplantation, TLOs are also known as bronchus-associated lymphoid tissue (BALT). Migration of lymphocytes into lung tissue to form BALT through HEVs and lymphocyte homing is directed in part by α4β1 integrin, VCAM-1 and LFA-1 (84) and is dependent on expression of IL-22 (78). Originally only thought to be associated with rejection, a murine study in which lungs were first into immunosuppressed recipients for 72 hours followed by re-transplantation into untreated allogenic recipient, resulted in long-term survival and was associated with presence of Tregs in close proximity to dendritic cells within the BALT (85). The importance of Tregs in inducing tolerance in this setting was validated by their depletion using CD25 monoclonal antibody in the initial 72-hour period during which tolerance was established (85). The presence of TLOs in mouse lung allografts can also, however, be associated with an increase in rejection and fibrosis (86).

TLOs have also been described in kidney allografts. In a non-human primate study, lymphoid aggregates were identified in kidney allografts by immunohistochemistry (87). These were few in numbers after transplant but increased in rejecting grafts (87). In line with the idea that lymphoid structures are detrimental to graft survival, two animals that had operational tolerance had these cellular infiltrates disappear after the during the first year post-transplant (87). However, the presence and function of Tregs were not assessed in these grafts. Similarly, a study involving a cohort of transplant patients who experienced chronic rejection, cellular infiltrates of T cells, B cells, and DCs were found in clusters with plasmablasts and plasma cells (82). Here, Tregs were not predictive of longevity of the grafts. The benefit of studying kidney transplant patients is the availability of protocol biopsies performed at certain centers allowing for availability of samples throughout the lifespan of the graft. From these biopsies, patients with subclinical rejection without FOXP3^+^ infiltrates in the graft had worse graft outcome than those with FOXP3^+^ infiltrates and those without subclinical rejection (88). The presence of FOXP3^+^ Tregs seemed to confer a graft outcome similar to that seen in those with a normal non-subclinical rejection biopsy (88). In a highly tolerant murine model, C57BL/6 mice received DBA/2 kidneys that developed operational tolerance, grafts were developed periarterial Treg-rich lymphoid structures, in which the Tregs expressed latency-associated peptide (LAP) and exhibited increased proliferation activity (89).

TLOs are a rich environment in which numerous immune cells have the opportunity to interact via cognate antigen-antigen receptor interactions, costimulation and checkpoint pathways, and cytokine signaling (89). In contrast to the prevailing view, in which TLO development is lymphotoxin-dependent, in this study TLOs formed in response to MHC class II disparity without the need for lymphotoxin pathways (89). These findings suggest that although TLO formation within allografts can be associated with both rejection and acceptance, the developmental pathway, localization and composition of these TLOs may be the driving factor that dictates their role in allograft outcome. Close and early interaction between Tregs and dendritic cells may be needed to prevent activation and proliferation of effector T cells, but if interaction occurs after effector T cells have experienced antigen and proliferated, the presence of Tregs is unlikely to be sufficient in controlling alloimmunity (90). Nevertheless, positioning of Tregs within TLOs – where they are likely to encounter many antigen-presenting cells – may be optimal for immune regulation. Furthermore, it has recently become apparent that Tregs are a heterogeneous population, and this may be one of the factors that determines their localization and function within TLOs and other allograft microenvironments.

## Multidimensional analytic approaches to exploring Treg heterogeneity in allografts

Originally, Treg subpopulations were difficult to parse apart, but recently technological breakthroughs have made high dimensional analysis of transcriptomes and protein expression at the single cell level possible. Techniques such as mass cytometry, single-cell (scRNA-seq), single nucleus RNA sequencing (snRNA-seq), and multiplexed imaging have enabled the investigation of Treg subpopulations, their differentiation pathways and phenotype, which would have otherwise been undetectable using conventional bulk tissue analysis. These techniques allow for further analysis into Treg status in different tissue types, including their activation status, epigenetic changes, and cytokine production.

scRNA-seq is a high dimensional technique used to analyze the transcriptome of single cells. By capturing all the RNA content of individual cells, scRNA-seq preserves the granularity and complexity of subpopulations that are lost in bulk RNA sequencing approaches. Usage of the entire sequenced transcriptome in scRNA-seq analysis allows for shifts in cellular differentiation pathways and expression profiles to be detected. Coupled with unsupervised dimensionality reduction analysis, cells with shared transcriptional profiles can be grouped into clusters using tools such as Seurat (91) and singleR (92); gene expression patterns can be compared between populations using heatmaps and algorithms such as DESeq2 to determine differential gene expression by utilizing negative binomial generalized linear models (93). Comparison of gene expression can be used to determine if two conditions or treatment modalities have an effect on the composition of cell population and if these cells exhibit changes in their gene expression. The usage of such analysis methods in the comparison of rejecting and stable or tolerant allografts may reveal differences in their immune cell composition that could reveal important mechanistic pathways.

Investigators have begun to use these approaches to identify novel Treg subpopulations, providing insights into the transcriptional regulatory networks governing Treg biology. Utilizing single cell transcriptomics and high dimensional cytometry has provided further insights into the heterogeneity and differentiation pathways of Treg populations. At the single-cell RNA transcript level, Tregs are commonly identified using FOXP3, CTLA4 and IL2RA gene expression (94, 95). However, since single-cell suspensions are needed for these techniques, spatial information on where cell populations of interest are distributed within the tissue is lost. Cells that need to be analyzed in the context of their neighbors require techniques that include spatial information. These techniques include spatial transcriptomics, in which the distribution of transcripts in tissue is visualized through imaging of hybridized fluorescent probes or recording of locations for sequencing-based approaches (96), albeit at low resolution. Protein-based detection approaches include imaging mass cytometry (IMC), multiplexed ion beam imaging by time-of-flight (MIBI-TOF), co-detection by indexing (CODEX) and cyclic Immunofluorescence (CycIF) (96). These approaches provide high-dimensional information on cellular transcriptomes or protein expression while maintaining the spatial data through mapping back information on to their histological locations (97). This is achieved through *in silico* processing which reconstructs the positions of all detected cells via gene expression providing spatial information that enables analysis of cell-cell interactions and visualization of cells within structures (96). These high dimensional techniques offer a more detailed view into the complexities of Tregs and the changes that environmental cues impose. With these and other methods, subpopulations of Tregs are beginning to be described which may provide the granularity needed to fully understand their phenotype and function in allograft tolerance, although it should be noted that these techniques have their limitations.

## Multidimensional data provides evidence for unique properties of tissue Tregs

Tregs are conventionally found in secondary lymphoid organs such as the spleen, lymph nodes, tonsils, and Peyer’s patches. However, distinct populations of Tregs have also been reported in non-immunological tissues (98). In comparison to circulating conventional Tregs, subsets of Tregs home and migrate into tissues where they further differentiate and develop specialized functions (10, 98–101). These Tregs are not a result of chronic inflammation or immune perturbation but have homeostatic functions that are specific to the needs of the tissue (100, 102). Compared to conventional Tregs that migrate through the lymphatics and remain in the lymph nodes, the presence of Tregs in tissue allows for localized immune modulation and a tailored response (103). A deeper understanding of Treg populations in normal tissues is likely to inform the study of Tregs in accepted and rejecting allografts.

Tregs have been identified in the visceral adipose tissue (VAT) in mice (100). VAT Tregs are not a result of TGF-β induced conversion of FOXP3^-^CD4^+^ cells to FOXP3^+^CD4^+^ cells, but are in fact thymocytes that migrate to VAT tissue early in life (100, 104, 105). VAT Tregs have been proposed as modulators of insulin resistance in obese mice (100), although in other studies they have been shown to fail at promoting insulin resistance in aged mice (106). In addition to VAT Tregs, the skin, which is the largest barrier surface in the body, has a unique microbiome that is required for self-tolerance (107). Perhaps not surprisingly, the skin has resident Tregs (108). Skin Tregs function to promote tolerance to commensal microbes, and are recruited rapidly in the neonate (109). Skin Tregs modulate the immune system and prevent skin inflammation (110, 111). Aside from maintaining homeostasis, skin Tregs also promote tissue repair, through transcription factor BATF (112), amphiregulin (AREG) and proenkephalin (PENK) (107). Similar to the skin, the intestine separates the external and internal environment and has its own microbiota. Intestinal Tregs have described as having multiple roles to ensure homeostasis; loss of intestinal Tregs can result in inflammation and inflammatory bowel diseases such as ulcerative colitis and Crohn’s disease (113). Intestinal Tregs were described as divided into three groups; GATA3^+^Helios^+^, RORγt^+^, RORγt^-^ Helios^-^ Tregs (114). These subpopulations of intestinal Tregs are likely the result of unique stimuli present in the intestine, since Treg expression of RORγt is modulated by the intestinal microbiota (115, 116). IL-33 signals through transforming growth factor (TGF)-β1 leading to the differentiation of Tregs and their accumulation in the intestine (117). These tissue Tregs are similar to conventional Tregs, but their specialized functions have made them suitable for their tissue microenvironment.

Differentiation of tissue specific Tregs varies with the tissue and is not fully understood. Tissue Tregs of the colon, VAT and skeletal muscle from mouse were analyzed and found that for tissue modifications many open coding regions were already accessible in the spleen, suggestive of a pre-differentiation event that primed the transition of Tregs from lymphoid to nonlymphoid organs (118). However, it should be noted that a shared pattern of open chromatin does not necessitate similar gene expression between cells; additional transcription factors drive specific gene expression and it was indeed found that a limited set of transcription factors regulated tissue Treg genes (118). Zemmour et al. used scRNA-seq and described a core of FOXP3 dependent transcripts that were uniformly expressed in Tregs that were under expressed or missing in conventional T cells (94). Described as modular, the authors propose that on top of the core Treg transcriptional program, additional transcripts are expressed as a function of differentiation and location (94). Secondly, they also found that TCR signaling intensity shaped activated Treg differentiation (94), suggesting that the presence of antigen in the microenvironment impacts upon tissue Treg development. Analysis of transcripts within the Treg population revealed a continuum of Treg states leading to fully differentiated tissue Tregs, which are characterized by tumor-necrosis factor receptor superfamily (TNFRSF) gene expression – specifically TNFRSF4, TNFRSF9 and TNFRSF18 – and expression of other non-lymphoid tissue-associated genes such as KLRG1, RORA, and ITGAE (10). Miragaia et al., used mouse skin and colon samples and comparison with nearby lymphoid tissue to reveal tissue-specific differentiation signatures. Originating from lymph nodes, colon and skin resident Tregs developed through a shared pathway with expression of GATA3, IL1RL1, TNFRSF4 and RORA; tissue specific gene expression was also evident, where skin Tregs expressed DGAT2 while colon Tregs expressed ITGA4 and GIMAP6 (10). Through trajectory analysis Luo et al., described two differentiation pathways originating from a common origin of CCR7^+^ cells (11). One pathway was described as CCR7^-^CCR4^mid/hi^ and had more suppressive capacity with enrichment in gene transcripts related to cell-cell adhesion and aggregation (11). The second pathway was described as CCR7^-^CXCR3^+^ and had higher proliferative capacity than pathway one and expressed glycolysis and tricarboxylic acid cycle transcripts (11). A chromatin accessibility comparison of VAT and skin Tregs with blood Tregs suggested a developmental trajectory of blood naïve Tregs to blood memory Tregs rather than to tissue Tregs (112). Combined, these data suggest that Tregs lie on a continuum of progressive adaptation to tissue and differentiation in response to their environment (10, 94). These tissue specific Tregs may be differentiated from conventional Tregs based on additional modules of gene expression, on top of the core regulatory program, that confer tissue-specific functions. Continued exploration of tissue Tregs may provide a basis for promoting Tregs function within allografts to promote tolerance.

## Single-cell characterization of Tregs in allograft tissue

As tissue resident Tregs have now been described to have specialized functions and do not appear to arise as a result of inflammation or perturbations to the local environment, the possibility arises that some allografts may also contain a homeostatic niche for similar Tregs independent of the alloimmune response. However, this notion stands in contrast to observations that the Treg content of allografts increases during rejection concomitantly with the infiltration of other T cell populations (48, 49, 55).

To resolve these questions, investigators have begun to apply high dimensional techniques to transplanted organs. In a murine cardiac transplant model analysis via a 37-parameter CyToF panel was applied to splenocytes. The authors described four Treg populations, two of which had higher expression of GITR and MHCI, where MHCI expression was associated with allograft rejection (119). These Treg clusters are denoted as GITR^mid^CD25^-^MHCI^lo^, GITR^lo^CD25^lo^MHCI^lo^, GITR^hi^CD25^-^MHCI^hi^ and GITR^hi^CD25^hi^MHCI^hi^. The proportion of GITR^mid^CD25^-^MHCI^lo^ and GITR^lo^CD25^lo^MHCI^lo^ cells was higher in no transplant control when compared to syngeneic and allogeneic day 5 post-transplant (119). Accordingly, GITR^hi^CD25^hi^MHCI^hi^ Tregs increased in proportion in allogeneic transplants at day 5 when compared to control (119). This change in Treg cluster proportions and expression of GITR and MHCI may have been due to chronic stimulation and activation of Tregs due to chronic antigen presentation.

In a paper by Liu and colleagues, two kidney transplant recipients were biopsied and the tissue was analyzed via scRNA-seq and compared to a published healthy kidney dataset (120). Annotation of the data revealed a sub-cluster of FOXP3-expressing cells within what the authors described as the natural killer T cell cluster. Unfortunately, further in-depth analysis on this cluster to determine if these are truly Tregs or whether other Treg populations exist was not performed, and the data are not publicly available (120). Li and colleagues analyzed liver samples and blood from 55 liver transplant patients using scRNAseq to characterize the immune landscape of the allograft (121). Interestingly, upon further analysis of a CTLA4^+^CD8^+^ T cell population, they also found a subset of CD4^+^CD8^+^FOXP3^+^ T cells. The authors performed pseudotime analysis, a method that estimates the order of cell differentiation in atemporal resolution without the need for prior knowledge (122) and suggested that CD8^+^ T cells infiltrated the graft and subsequently developed regulatory function through increasing expression of both CD4 and FOXP3 (121). Any conclusions made from this observation must account for the fact that RNA expression does not correspond directly with protein level expression, so although levels of CD4 and CD8 can be detected at the RNA level, this may not translate to the protein level so this cluster may in fact express only one of the co-receptors.

In addition to gene expression, scRNA-seq methodology also allows for capturing T cell receptor (TCR) sequences when the RNA is analyzed from the 5’ end instead of the 3’ end of the transcript, given the location of the variable sequence of the TCR is nearer the 5’ end. As TCR signaling is required for Treg suppression and extravasation into target tissue as well as into secondary lymphoid organs, analysis of TCR clonality and repertoire may further our understanding of Treg homing and retention in allografts. In this regard, TCR analysis of peripheral blood and liver grafts of 7 liver transplant recipients that were either stable, experiencing subclinical rejection or acute cellular rejection was performed pre-transplant and at 12- and 36-months post-transplant (123). TCR clonality did not increase in peripheral blood, but clonality of donor reactive CD4^+^ and Tregs did increase in the liver allograft (123). The TCR repertoires differed between peripheral blood and liver allograft and showed only limited overlap with decreased frequency post-transplant at both 12 months and 36 months (123). No change in TCR repertoire was noted when focusing on the bulk population, but donor reactive GARP^+^ Tregs displayed significantly higher clonal repertoire compared to control (123). Tracking of Treg clones in the peripheral blood at 12 months found that the although the frequency of GARP^+^ Tregs in blood was 13.95%, 47.73% of the trackable hepatic Tregs were alloreactive in the blood, pointing to an accumulation of donor reactive cells in the graft. This may be indicative of TCR signaling-driven Treg differentiation into tissue-resident cells as previously suggested (94).

## Exploration of putative Treg populations in published allograft datasets

**Figure 2:**
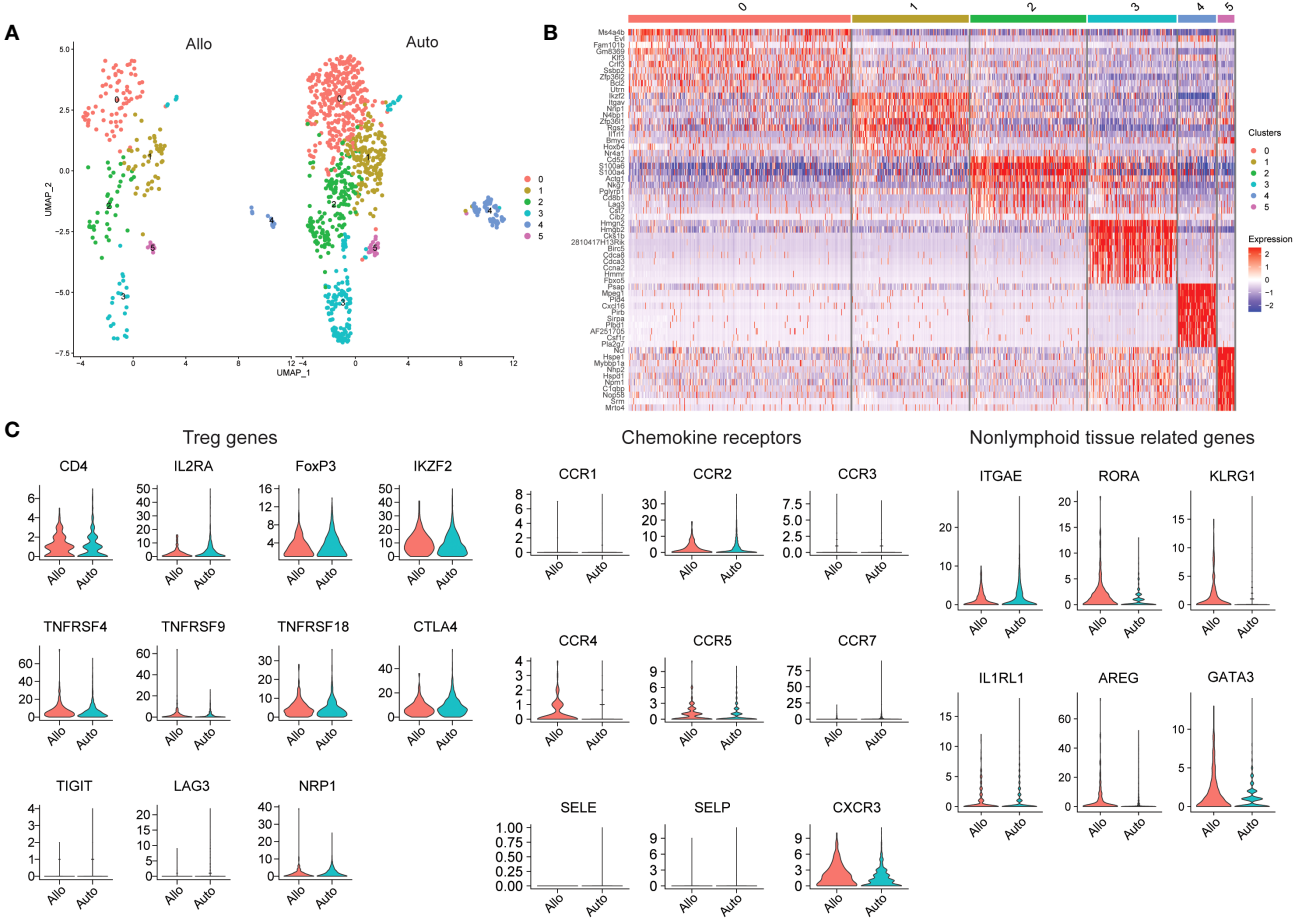
Analysis of Tregs in Mouse Islet Allografts and Syngeneic Grafts (GSE198865). **(A)** Dimensional reduction plot of allo-graft and auto-graft cells with FOXP3 > 0. Cells are separated into six clusters. **(B)** Heatmap indicating the different cell clusters of FOXP3 expressing cells, shows six clusters. Cluster 0 expressing MS4A4B, FAM101B, KLF3 and BCL2, cluster 1 expressing IKZF2 and IL1RL1, cluster 2 expressing CD8b1, and cluster 3 expressing high levels of cell cycle genes. Genes expressed in cluster 4 ranged from hematopoietic differentiation to receptor-type transmembrane glycoproteins. Cluster 5 expressed many heat shock proteins, along with podocin and ribonucleoprotein. **(C)** Violin plots depicting genes relating to Tregs (CD4, IL2RA, FOXP3, IKZF2, TNFRSF4, TNFRSF9, TNFRSF18, CTLA4, TIGIT, LAG3, and NRP1), chemokine receptors (CCR1, CCR2, CCR3, CCR4, CCR5, CCR7, SELE, SELP, and CXCR3) and non-lymphoid tissue related genes (ITGAE, RORA, KLRG1, IL1RL1, AREG, and GATA3).

In order to characterize allograft-resident Tregs in greater detail, we performed a secondary analysis of two publicly available datasets from previously published work. In one study, donor islets from C57BL/6 mice were transplanted into either C57BL/6 mice (isograft) or into BALB/c mice (allograft) under the kidney capsule and analyzed by scRNAseq at 7 days post-transplant (124). Tregs were defined based on FOXP3 and IL2RA expression. A higher proportion of Tregs was noted in allografts compared to isografts (124). The authors did identify a population of Tregs in the grafts, but further analysis of the Treg population to determine similarities and differences between allogeneic and syngeneic grafts was not performed (124). After obtaining the data via the Gene Expression Omnibus database (GSE198865), we performed further analysis on FOXP3 expressing cells (**Figure 2**). Six subclusters were identified based on FOXP3 expression were found, with cluster 0 expressing MS4A4B, FAM101B, KLF3 and BCL2. Cluster 1 had both IKZF2, which encodes for Helios, and IL1RL1 which encodes for ST2, receptor for IL33 (**Figure 2B**). Of the other four clusters, one cluster expressed CD8b1, therefore possibly representing a CD8^+^ Treg population, and another expressed high levels of cell cycle genes (**Figure 2B**). Genes expressed in cluster 4 ranged from hematopoietic differentiation to receptor-type transmembrane glycoproteins. Cluster 5 expressed many heat shock proteins, along with podocin and ribonucleoprotein (**Figure 2B**). These two clusters with a wide variety of genes underscore the importance, discussed above, of sample processing and quality control, as tissue processing to generate suspensions of single cells often generates spurious stress transcripts (125). Proper filtering of data will ensure removal of doublets and low quality cells, the presence of which will lead to detection of aberrant gene expression (126, 127).

We also explored the gene expression of a select number of markers to determine Treg activation (TNFRSF4, TNFRSF18, TIGIT, and LAG3), chemokine receptors and tissue Treg related genes (**Figure 2C**). Looking at the whole expression levels of either allo- or isografts, most Treg related markers genes similar between the grafts with the exception of IL2RA (CD25) and LAG3, which were higher in isografts. The distribution of NRP1 expression in allograft Tregs was wide, but generally NRP1 expression was higher in isograft Tregs, suggesting that perhaps most of the FOXP3-expressing cells in isografts were tTregs. Most of the chemokine receptors and selectins were expressed at a very low level in Tregs from both types of graft. CCR2, CCR3 and CCR7 transcripts seemed to be higher in isograft Tregs while CCR4 and CXCR3 transcripts were instead higher in allograft Tregs. Treg CCR5 expression was for the most part similar between the graft types. With the nonlymphoid-related markers (ITGAE, RORA, KLRG1, IL1RL1, AREG, and GATA3); RORA, KLRG1, AREG and GATA3 were higher in allograft Tregs, while ITGAE and IL1RL1 were instead higher in isograft Tregs. The combination of CCR4 and AREG expression may suggest that FOXP3-expressing cells are recruited and activated in allografts to promote repair.

**Figure 3:**
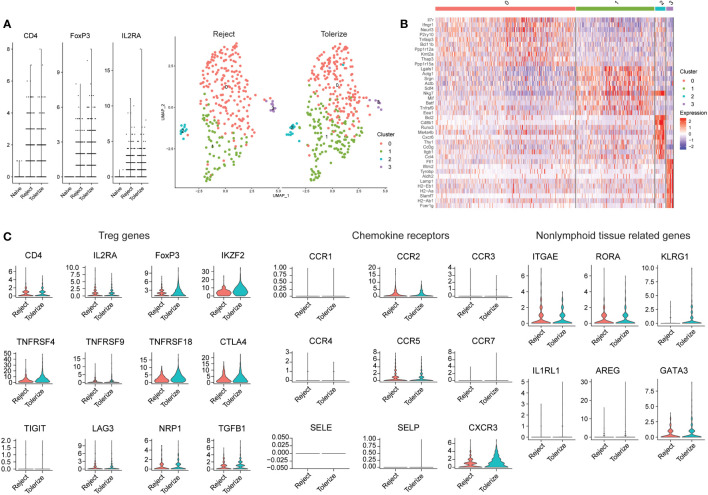
Analysis of Tregs in rejecting and tolerant mouse kidney allografts (GSE157292). **(A)** Violin plot prior to selecting on FOXP3 expressing cells, showing that many cells were from rejected and tolerized grafts while naïve grafts had no/low expression of FOXP3 (left). Dimensional reduction plot of rejected and tolerized grafts from FOXP3 > 0 expressing cells (right). **(B)** Heatmap indicating the different cell clusters of FOXP3 expressing cells, showing four clusters. Characterized by high expression of IL7R and IFNGR1 in cluster 0, LGALS1 and SRGN in cluster 1, BCL2 and CD8 in cluster 2, BCL2 and CD8 in cluster 3 and a few histocompatibility molecules in cluster 3. **(C)** Violin plots depicting genes relating to Treg (CD4, IL2RA, FOXP3, IKZF2, TNFRSF4, TNFRSF9, TNFRSF18, CTLA4, TIGIT, LAG3, NRP1, and TGFB1), chemokine receptors (CCR1, CCR2, CCR3, CCR4, CCR5, CCR7, SELE, SELP, and CXCR3) and nonlymphoid tissue-related genes (ITGAE, RORA, KLRG1, IL1RL1, AREG, and GATA3.

In another murine study analyzing allografts using scRNAseq, kidneys from 6-7 week old BALB/c mice were transplanted into bilaterally nephrectomised B6 male recipients and macrophages were found to infiltrate the graft (128). Kidney allograft-infiltrating myeloid cells differentiated from monocytes to proinflammatory macrophages by expression of Axl, potentially through interactions with the transcription factor C/EBPβ (128). Kidney allograft tolerance was induced by infusion of donor apoptotic cells (128). The authors did not specifically investigate Tregs in tolerance and rejection but shared their data on Gene Expression Omnibus (GSE157292). We were able to select on cells with FOXP3 > 0 for further analysis (**Figure 3**). In general, a higher expression of FOXP3 can be detected in the Tregs of tolerized grafts compared to rejected grafts, but no large differences in the proportions of Treg subclusters was seen (**Figure 3A**). Both rejected and tolerized grafts had populations of FOXP3 expressing cells, but this population was missing from kidneys in naïve animals. Four distinct subpopulations of FOXP3 expressing cells were found, characterized by high expression of IL7R and IFNGR1 in cluster 0, LGALS1 and SRGN in cluster 1, BCL2 and CD8 in cluster 2, and a few histocompatibility molecules in cluster 3 (**Figure 3B**).

Analysis of the gene expression of selected markers show that for the most part, the expression of Treg related genes is similar between rejected and tolerized grafts with the exception of FOXP3, which was higher in the Tregs of tolerized grafts (**Figure 3C**). A similar higher expression of IKZF2, also known as Helios and NRP1 – neuropilin-1 was higher in the tolerized grafts. These Tregs may be more activated as indicated through the expression of TNFRSF4 and TNFRSF18. Many of the chemokine receptors are also of similar values and expression between Tregs in the two types of graft, except for CXCR3 which was found to be more highly expressed in the Tregs of tolerized grafts. Nonlymphoid -related markers such as KLRG1 and GATA3 were higher in the Tregs of tolerized grafts while ITGAE, RORA were approximately of the same level of expression in the Tregs of both types of grafts. A slightly higher mRNA expression of AREG was also detected in the Tregs of tolerized grafts although the difference was small. These gene expression patterns seem to indicate that for the most part, similar expression levels of genes were seen in Tregs between the two types of grafts but additional activation of the Tregs, changes to trafficking molecules and expression of repair molecule amphiregulin indicate that the Tregs in tolerized grafts may have taken on additional roles that perhaps drove tolerance. How these observations correspond to functional differences remains to be investigated. Still, the distribution of intragraft FOXP3 expressing cells into five distinct clusters indicates that Tregs are not a homogenous population and that this must be taken into account when analyzing intragraft Tregs.

It should be noted that these analyses are only the start of understanding Treg phenotype in transplanted organs. FOXP3 expression does not equate strictly to Treg identity and has also been described in CD4^+^CD25^−^T cells, and cancer cells (56–59). Therefore, when FOXP3 expression greater than 0 is used to identify Tregs, it must be borne in mind that cells with this property instead represent a mixture of related cell types that share in the expression of FOXP3. Also, although analysis of TCR sequences is now possible, allowing inferences to be made about the expansion of potentially alloreactive Treg populations, these currently remain only inferences because our ability to identify cognate peptide-MHC ligands from TCR sequences is limited. Furthermore, caution must be used in interpreting these data, as scRNA-seq datasets include noise from doublets and dying cells which can result in false signals (126, 127). In a similar vein, false-positive transcripts can be detected due to poor filtering of data leading to erroneous differential gene expression analysis (129). Batch effects, where expression of genes in one batch uniformly differs from another batch, can also arise in scRNA-seq data. Identifying and correcting for such batch effects is essential for robust data interpretation (130). As high dimensional techniques become more commonplace, understanding of their advantages and limitations is essential to proper interpretation of the data. Detection of irrelevant genes is a possibility when proper quality control and processing of samples are not put into practice. Ultimately, hypothesis-driven functional validation studies will be required to confirm these observations.

## Conclusions

With the development of high dimensional techniques, detecting and distinguishing small gene expression differences and rare cell populations is now possible. This has enabled identification of Treg subpopulations, which may lead to better understanding of the role of the allograft microenvironment in the differentiation and function of Tregs. As these techniques become more routine, the limitations noted above must be borne in mind. Analysis pipelines need to be carefully validated to ensure high quality data are properly analyzed. In addition to single-cell techniques, recent advances in high-dimensional spatial biology approaches can provide information on Treg cellular context and its relationship to tissue structures, to aid in the understanding of the role of Tregs in allograft longevity.

A large body of evidence supports the concept that intragraft Tregs are critical for allograft acceptance. Translating the current understanding of intragraft Tregs from gene profiles derived from scRNA-seq data to protein-level validation will be an important next step as gene expression does not correlate directly to protein expression. Usage of newer high dimensional transcriptomic methods that combine protein analysis with spatial locations such as IMC can be a step to understanding the formation and structure of allograft infiltrates and their phenotype including Tregs.

Finally, the advent of ex vivo allograft perfusion systems opens up the possibility for directed graft modulation using Tregs (131) and other immunoregulatory approaches, such as allograft genetic engineering (132). An analysis of the properties of Tregs in accepted or stable allografts may inform the rational design of these therapies. Since systemic infusion of Tregs was shown to be feasible without adverse effects in a phase I/II kidney transplant trial (75), optimization of an allograft-directed approach could be readily clinically translatable. Hence, a better understanding of Treg transcriptional states within allografts may lead to a fuller understanding of how to facilitate the induction of operational tolerance, improving outcomes for thousands of patients facing the prospect of organ transplantation.

## Methods supplement

Public data from the Gene Expression Omnibus (GSE198865) and (GSE157292) were selected for Treg analysis. Samples were selected on mitochondrial gene content less than 10% for all samples. For samples from GSE198865, nFeature(number of genes in a cell) was selected between 200 and 5000. For samples from GSE157292, naïve kidney samples were selected between 500 to 2000 genes per cell, for both rejected and tolerized kidney samples, 200 to 3000 genes per cell were selected. Tregs were defined as cells with FOXP3 RNA > 0 prior to SCT integration(1, 2). Due to low cell count (94), all cells in the smallest sample were considered when weighing anchors for GSE157292. Distinct clusters of Tregs were displayed on a Uniform Manifold Approximation and Projection (UMAP) plot and the top 10 differentially expressed genes in each cluster were shown in a heatmap using DoHeatmap (91). Based on the literature, CD4, IL2RA (CD25), FOXP3, IKZF2 (Helios), TNFRSF4 (OX40), TNFRSF9 (4-1BB), TNFRSF18 (GITR), TIGIT, LAG3 and NRP1 (neuropilin 1) were selected as Treg genes, and expression level was shown using violin plot for the different types of grafts. Similarly, chemokine receptors, CCR1, CCR2, CCR3, CCR4, CCR5, CCR7, selectin E, selectin P, and CXCR3 were also depicted in a violin plot. To determine if nonlymphoid tissue-related genes were expressed differentially in the grafts, ITGAE (CD103), RORA, KLRG1, IL1RL1 (ST2), AREG (amphiregulin), and GATA3 genes were also displayed as violin plots.

## Author contributions

KB: Conceptualization, Data curation, Formal Analysis, Writing – original draft, Writing – review & editing. SM: Conceptualization, Data curation, Formal Analysis, Writing – review & editing. BL: Data curation, Writing – review & editing. SJ: Conceptualization, Funding acquisition, Supervision, Writing – review & editing.
